# Synthetic lethality of combined ULK1 defection and p53 restoration induce pyroptosis by directly upregulating GSDME transcription and cleavage activation through ROS/NLRP3 signaling

**DOI:** 10.1186/s13046-024-03168-8

**Published:** 2024-08-30

**Authors:** Wei Chen, Kai-Bin Yang, Yuan-Zhe Zhang, Zai-Shan Lin, Jin-Wei Chen, Si-Fan Qi, Chen-Fei Wu, Gong-Kan Feng, Da-Jun Yang, Ming Chen, Xiao-Feng Zhu, Xuan Li

**Affiliations:** 1 Department of Radiation Oncology, Sun Yat-sen University Cancer Center; State Key Laboratory of Oncology in South China, Collaborative Innovation Center for Cancer Medicine; Guangdong Key Laboratory of Nasopharyngeal Carcinoma Diagnosis and Therapy, Guangzhou, China; 2 State Key Laboratory of Oncology in South China, Collaborative Innovation Center for Cancer Medicine, Sun Yat-sen University Cancer Center, 651 Dongfeng Road East, Guangzhou, China; 3 Zhongshan School of Medicine, Sun Yat-Sen University, Guangzhou, China; 4 United Laboratory of Frontier Radiotherapy Technology of Sun Yat-sen University & Chinese Academy of Sciences Ion Medical Technology Co., Ltd, Guangzhou, China

**Keywords:** MDM2 inhibitor, Mitophagy, Pyroptosis, Reactive oxygen species, TP53, ULK1

## Abstract

**Background:**

High expression of ubiquitin ligase MDM2 is a primary cause of p53 inactivation in many tumors, making it a promising therapeutic target. However, MDM2 inhibitors have failed in clinical trials due to p53-induced feedback that enhances MDM2 expression. This underscores the urgent need to find an effective adaptive genotype or combination of targets.

**Methods:**

Kinome-wide CRISPR/Cas9 knockout screen was performed to identify genes that modulate the response to MDM2 inhibitor using TP53 wild type cancer cells and found ULK1 as a candidate. The MTT cell viability assay, flow cytometry and LDH assay were conducted to evaluate the activation of pyroptosis and the synthetic lethality effects of combining ULK1 depletion with p53 activation. Dual-luciferase reporter assay and ChIP-qPCR were performed to confirm that p53 directly mediates the transcription of GSDME and to identify the binding region of p53 in the promoter of GSDME. ULK1 knockout / overexpression cells were constructed to investigate the functional role of ULK1 both in *vitro* and in *vivo*. The mechanism of ULK1 depletion to activate GSMDE was mainly investigated by qPCR, western blot and ELISA.

**Results:**

By using high-throughput screening, we identified ULK1 as a synthetic lethal gene for the MDM2 inhibitor APG115. It was determined that deletion of ULK1 significantly increased the sensitivity, with cells undergoing typical pyroptosis. Mechanistically, p53 promote pyroptosis initiation by directly mediating GSDME transcription that induce basal-level pyroptosis. Moreover, ULK1 depletion reduces mitophagy, resulting in the accumulation of damaged mitochondria and subsequent increasing of reactive oxygen species (ROS). This in turn cleaves and activates GSDME via the NLRP3-Caspase inflammatory signaling axis. The molecular cascade makes ULK1 act as a crucial regulator of pyroptosis initiation mediated by p53 activation cells. Besides, mitophagy is enhanced in platinum-resistant tumors, and ULK1 depletion/p53 activation has a synergistic lethal effect on these tumors, inducing pyroptosis through GSDME directly.

**Conclusion:**

Our research demonstrates that ULK1 deficiency can synergize with MDM2 inhibitors to induce pyroptosis. p53 plays a direct role in activating GSDME transcription, while ULK1 deficiency triggers upregulation of the ROS-NLRP3 signaling pathway, leading to GSDME cleavage and activation. These findings underscore the pivotal role of p53 in determining pyroptosis and provide new avenues for the clinical application of p53 restoration therapies, as well as suggesting potential combination strategies.

**Supplementary Information:**

The online version contains supplementary material available at 10.1186/s13046-024-03168-8.

## Background

Tumor protein p53 (TP53) is a central determinant of cellular fate, orchestrating cellular processes such as cell cycle arrest and the activation of programmed cell death pathways through direct transcriptional activation [1]. P53 is inactive in the vast majority of tumors. Although the proportion of tumors with wild-type p53 genes is as high as 50%, the ubiquitin ligase MDM2 mediates its ubiquitination and subsequent degradation through the proteasome pathway, ultimately leading to functional deficiency of the p53 protein. Inhibiting MDM2 activity to restore p53 function is highly anticipated as a clinical treatment strategy for this subset of wild-type tumors. However, existing MDM2 inhibitors have limited clinical application windows and considerable side effects due to mechanisms such as feedback activation of MDM2 [2]. The outcomes of clinical trials have been unsatisfactory, highlighting the urgent need to identify adjunctive therapeutic targets to optimize treatment strategies [3].

Pyroptosis is a newly identified form of programmed cell death that is distinct from apoptosis, and its occurrence requires the cleavage and activation of a class of GSDM family proteins by inflammatory caspases. GSDM cleavage releases the N-terminal domain of the protein to form pore-forming oligomers that insert into the cell membrane, leading to lytic cell death [4]. The expression of GSDM family proteins is a critical determinant of pyroptosis activation in cells [5, 6]. Previous studies have investigated the regulation of the expression of GSDM family genes within cells through multiple stages and from multiple perspectives, including DNA methylation, transcriptional regulation, and protein stability control [6–9]. The results of these studies indicate that inactivation or low expression of GSDM family members in tumors is a key factor contributing to tumor progression and therapeutic resistance. The activation of inflammasome pathway is a crucial trigger for pyroptosis. However, previous studies have focused mainly on the role of inflammasome pathway in mononuclear phagocytes [10, 11], and whether it participate in epithelium-derived cells during pyroptosis remains unclear.

In our studies, we identified ULK1 as a synthetic lethal gene that enhances cell sensitivity to MDM2 inhibitors through a CRISPR kinase library screen. Interestingly, we found that combining ULK1 deficiency with p53 activation can induce typical pyroptosis in cells. Further mechanistic studies revealed that p53 can directly bind to the GSDME promoter region to mediate its transcriptional activation. On the other hand, ULK1 deficiency inhibits mitophagy, leading to the accumulation of damaged mitochondria and increased ROS release, which further activates the NLRP3-Caspase3/8 signaling axis and mediates the cleavage and activation of GSDME. This study suggests that targeting ULK1 and activating p53 can synergistically reverse cisplatin resistance in cancer cells, providing a new direction for the clinical treatment of tumors by restoring p53 function.

## Methods

### Cell culture

Human embryonic kidney cells (HEK293T) were purchased from American Type Culture Collection (ATCC). Human lung adenocarcinoma cells (A549) were generously provided by Dr. Ming CHEN at Sun Yat-Sen University Cancer Center. Human ovarian cancer cells (A2780 and TOV21G) were generously provided by Dr. Xiaofeng Zhu at Sun Yat-Sen University Cancer Center. HEK293T and A549 cells were grown in DMEM (C11995500BT, Gibco, Grand Island, NY, USA) supplemented with 10% FBS (10,099,141, Gibco, Grand Island, NY, USA), while A2780 and TOV21G cells were grown in RPMI 1640 medium (C11875500BT, Gibco, Grand Island, NY, USA) containing 10% FBS (10,099,141, Gibco, Grand Island, NY, USA). All cell lines were cultured in a 37°C incubator with a humidified 5% CO2 atmosphere to mimic physiological conditions. Penicillin‒streptomycin (100 IU/ml) (MP Biomedicals, Irvine, CA, USA) was added as necessary. All experiments were performed in cells at 70–80% confluence, ensuring that cells are in an optimal growth phase.

### Reagents and antibodies

APG-115 was generously donated by Professor Yang Dajun [12]. baf A1 (S1413), CHX (S7418), NAC (S1623), idasanutlin (S7205), MRT68921 (S7949), cisplatin (S1166) and CQ (S6999) were purchased from Selleckchem (Houston, TX, USA). Penicillin‒streptomycin (0916704) and puromycin (02190146) were purchased from MP Biomedicals (Irvine, CA, USA). TNF-alpha (HY-P7058) was purchased from MedChemExpress (Monmouth Junction, NJ, USA). The following antibodies were used in our research. The anti-SQSTM1/p62 (sc-28359) antibody was purchased from Santa Cruz Biotechnology (Dallas, TX, USA), and the anti-LC3 (NB100-2220) antibody was purchased from Novus Biologicals (Centennial, CO, USA). Antibodies against DFNA5/GSDME (13,075–1-AP), GAPDH (60,004–1-Ig), TP53 (60,283–2-Ig), MDM2 (66,511–1-Ig), p21 (10,355–1-AP), BAX (50,599–2-Ig), BAD (10,435–1-AP), Caspase3/p17/p19 (66,470–2-Ig), ATG5 (10,181–2-AP), ATG7 (10,088–2-AP), FUNDC1 (28,519–1-AP), PARK2/Parkin (14,060–1-AP), Tim23 (11,123–1-AP), HSP60 (15,282–1-AP), and NLRP3 (19,771–1-AP), as well as horseradish peroxidase (HRP)-conjugated goat anti-mouse IgG (SA00001-1) and HRP-conjugated goat anti-rabbit IgG (SA00001-2), were purchased from Proteintech (Rosemont, IL, USA). Antibodies against ULK1 (8054), TOMM20 (42,406), cleaved caspase 1 (4199), cleaved caspase 8 (9496) and cleaved IL1B (63,124) were purchased from Cell Signaling Technology (Danvers, MA, USA).

### Plasmids

The human full-length TP53 cDNA (HG10182-G, Sino Biological, Beijing, China) was cloned and inserted into the pSIN-EF2-puro vector (P40791, MiaoLingBio, Wuhan, China) between the BamHI and EcoRI restriction sites. The promoter regions of GSMDE were cloned and inserted into the pGL3-basic vector (E1751, Promega, Madison, WI, USA) between the HindIII and XhoI restriction sites. The ULK1 sequence was cloned and inserted into the pcDNA3.1( +) vector (V79020, Invitrogen, Carlsbad, CA, USA). To rescue/restore the expression of ULK1 in ULK1 knockout cells, synonymous mutations in ULK1 were generated by using specific primers, PrimeSTAR Max DNA Polymerase (Takara, R045B) and a ClonExpress II One Step Cloning Kit (Vazyme, C112). All newly constructed plasmids were verified by DNA sequencing. All plasmids were transfected into cell lines with Lipofectamine 3000 (Invitrogen, LC3000015) and Opti-MEM (Gibco, 31,985,070) according to the manufacturer's instructions.

### Kinome-wide CRISPR knockout screens

The kinome-wide CRISPR knockout screens were employed to systematically identify kinases that play critical roles in the cellular response to APG-115 treatment. The sgRNA pool consisted of 5170 sgRNA sequences with 10 sgRNAs per kinase (corresponding to 507 target genes and 100 control sgRNA sequences) and was synthesized by Synbio Technologies. After synthesis, the single-stranded sgRNA pool was amplified to obtain a double-stranded gRNA pool using PCR with universal primers and a high-fidelity enzyme kit. The PCR products were purified via agarose gel electrophoresis and gel extraction. The purified PCR products were then ligated into enzyme-digested plenti-CRISPR-V2 vectors to generate the sgRNA library. A2780 cells (1.4 × 10^8^) were transduced with the sgRNA library at a multiplicity of infection of 0.3. Then, the stably transduced cells were selected with 2 µg/ml puromycin, and 4 × 10^7^ cells were passaged every 24–48 h at a density of 2 × 10^6^ cells per 10 cm dish in A2780 growth medium for the duration of the screen. On the 7th day after puromycin selection, 8 × 10^7^ cells were treated with or without 32 µM APG-115 for 5 days. Then, 1 × 10^7^ cells were collected from the surviving population of APG-115-treated cells and an endpoint-matched untreated population.

The abundance of sgRNAs was determined by high-throughput sequencing. The data were analyzed by the software MAGeCK and the R package “MAGeCKFlute” [13]. To ensure the reliability of our findings, we established specific criteria for the selection of sgRNAs targeting kinases. Only those kinases with at least three “neg|goodsgrna” were included. Additionally, we required that these sgRNAs had a read count greater than 100 in the input group to ensure they were sufficiently represented in our sequencing data. Kinases meeting these criteria were deemed reliable and were subsequently used for result presentation and further validation studies. The kinases corresponding to sgRNA sequences with a decreased abundance in the drug-treated group compared to the control group were considered potentially sensitizing kinases upon deletion or inhibition in cells.

### Cell viability assays

Cells were seeded in a 96-well plate with 3 replicate wells per group and incubated overnight at 37°C in a cell culture incubator. Then, the cells were treated with the indicated drugs for 72 h. Subsequently, MTT was added to each well, and the plates were incubated at 37°C for 4 h. Afterward, the supernatant was removed, and the formazan crystals were dissolved in 200 µl of DMSO. Finally, the absorbance at 570 nm was measured by a microplate reader (Epoch Biotek, USA) to estimate the cell viability.

### Cell proliferation assay

Twenty-four hours after transfection, cells were seeded in a 96-well plate with 3 replicate wells (500 cells per well). The cells were counted every day by MTT assay over 5 days.

### Colony formation assay

Twenty-four hours after transfection, cells were seeded in 6-well plates (1000 cells per well). Fresh medium was added to the plates every 3 days. After incubation for 1–2 weeks, cells were fixed with 4% paraformaldehyde at room temperature for 10 min and stained with 0.5% crystal violet at room temperature for 60 min. Then the 6-well plates were scanned to observe.

### CRISPR-Cas9-mediated gene knockout

For validation of ULK1 expression after the initial screen, the following sgRNA sequences were designed and cloned to generate ULK1 knockout cells:

sgRNA1: GGGCTGAACACAGCTCCCAG.

sgRNA2: TTCACAGCATCACTGCCGAG.

The sgAAV1 sequence (GTCCCCTCCACCCCACAGTG) was used as a negative control.

Lentiviruses expressing wild-type Cas9 and sgRNAs were produced in the HEK-293T cell line. Cells were virally infected to express Cas9 and the sgRNAs to generate stable cell lines. Subsequently, the transduced cell lines were identified by puromycin selection for 3 days and seeded at a low density in a 96-well plate to obtain individual clones. The ULK1 knockout clones were identified through immunoblotting.

### Western blotting

Cells were washed with cold PBS and lysed in radioimmunoprecipitation assay (RIPA) lysis buffer (Cell Signaling Technology, 9806) supplemented with phenylmethanesulfonyl fluoride immediately before use. After centrifugation at 12,000 × g for 15 min at 4°C, the supernatant was collected, and the protein concentration was measured using a Pierce BCA Protein Assay Kit (Thermo, 23,227). Equal amounts of proteins were separated on sodium dodecyl sulfate (SDS)‒polyacrylamide gels and transferred to polyvinylidene fluoride (PVDF) membranes (Millipore, IPVH00010). After incubation with primary antibodies at 4°C overnight, the membranes were incubated with the corresponding HRP-conjugated secondary antibodies for 2 h at room temperature. Finally, chemiluminescence signals were detected with Clarity Western ECL Substrate (Bio-Rad, 1,705,061). The immunoblotting data was quantified with the ImageJ software (1.54g).

### Flow cytometry

To determine the proportion of Annexin V-fluorescein isothiocyanate (FITC)-positive cells, an Annexin V-FITC cell apoptosis detection kit (Beyotime, C1062S) was used, and the samples were analyzed according to the manufacturer’s instructions. Cells in the logarithmic growth phase were treated with drugs. After a certain period, the culture medium was collected into a centrifuge tube. The cells were detached from the culture dish using trypsin and collected into the same centrifuge tube. washed once with ice-cold PBS, then the cell pellet was resuspended in 195 µl of Annexin V-FITC binding solution. To this cell suspension, 5 μl of Annexin V-FITC and 10 μl of PI staining solution were added, and the sample was gently mixed and incubated at room temperature (20–25°C) in the dark for 10–20 min. Subsequently, the sample was placed on ice, and the fluorescence of the cells was evaluated in the FITC channel and the PI channel using a flow cytometer as soon as possible. 2',7'-Dichlorodihydrofluorescein diacetate (DCFH-DA; MCE, 4091–99-0), a ROS-sensitive fluorogenic probe, was used to measure intracellular ROS levels. Specifically, cells were incubated with 5 µM DCFH-DA in PBS in the dark for 30 min at 37°C. Following staining, the cells were harvested with 0.05% trypsin–EDTA solution, rinsed with PBS, resuspended in fresh medium, and immediately analyzed with a flow cytometer.

### Lactate dehydrogenase activity assay

The lactate dehydrogenase cytotoxicity assay kit was purchased from Beyotime (C0016). According to the kit instructions, cells in the logarithmic growth phase were seeded into a 96-well plate and treated with different concentrations of APG-115. Additional wells were reserved for culture medium without cells (background blank control wells) and untreated control cells (sample maximum enzyme activity control wells). One hour before the scheduled detection time, 10% of the volume of LDH release reagent was added to the sample maximum enzyme activity control wells. After the LDH release reagent was added, the plate was gently mixed, incubated at 37°C until the scheduled detection time, and then centrifuged at 400 × g for 5 min. Then, 120 μl of supernatant from each well was transferred to the corresponding well in a new 96-well plate, and 60 μl of LDH detection working solution was added to each well of this plate. After incubation at room temperature in the dark for 30 min, the absorbance was measured at 490 nm.

### Quantitative reverse transcription–PCR (qRT–PCR)

Total RNA was extracted from cultured cells using an EZ-press RNA Purification Kit (EZBioscience, B0004D), and reverse transcription was performed using a PrimeScript RT Reagent Kit with gDNA Eraser (Takara, RR047B). Real-time PCR was performed with SYBR® Premix Ex Taq™ II (Takara, RR820B) on a Light Cycler 480 instrument (Roche Diagnostics) following the manufacturer’s instructions. All genes were assayed in triplicate. The primers used to amplify GSDME were 5'-GGTCCTGTGCGTTTTGACAC-3' (forward) and 5'-CACGTTGGAGTCCTTGGTGA-3' (reverse). The primers used to amplify GSDMA were 5'-TGTTGGGGACGTACACGAAG-3' (forward) and 5'-TTCAAGTGCGAGCTCAAGGT-3' (reverse). The primers used to amplify GSDMB were 5'-TCCTGATTTCCGGGGAGCTA-3' (forward) and 5'-GAATTCGTGCCTCAGGGTCA-3' (reverse). The primers used to amplify GSDMC were 5'-GGAAGCAAAGACCTGACACCT-3' (forward) and 5'-GGAACGGTCCTGTCACAACA-3' (reverse). The primers used to amplify GSDMD were 5'-GAAGCCCTCAAGCTCATGGT-3' (forward) and 5'-GTCTGCCAGGTGTTAGGGTC-3' (reverse). The primers used to amplify GAPDH were 5'-AAGCCTGCCGGTGACTAAC-3' (forward) and 5'-GTTAAAAGCAGCCCTGGTGAC-3' (reverse). The primers used to amplify NLRP3 were 5'-GGAGGAGGACTTCGTGCAAA-3' (forward) and 5'-CCCGGCAAAAACTGGAAGTG-3' (reverse). The primers used to amplify IL1B were 5'-CCCTAAACAGATGAAGTGCTCC-3' (forward) and 5'-ATCTTCCTCAGCTTGTCCATG-3' (reverse). The primers used to amplify caspase 1 were 5'-TCGGCAGAGATTTATCCA-3' (forward) and 5'-GTTCTTCTAGGAATACTGTCAA-3' (reverse). The primers used to amplify caspase 8 were 5'-CCTTGGGAATATTGAGATTATATTCTCC-3' (forward) and 5'-ATAGCACCATCAATCAGAAGGGAAGACAAG-3' (reverse).

### Dual-luciferase reporter assay

The dual-luciferase reporter assay was performed to evaluate the transcriptional activity of the GSDME. The assay was conducted using the Dual-Luciferase Reporter Assay System (E1910, Promega, Madison, WI, USA) according to the manufacturer’s protocol. HEK-293T cells were seeded in 24-well plates at a density of 4 × 10^4^ cells per well and were then co-transfected with 0.5 µg of firefly luciferase reporter plasmid containing the promoter region of GSDME and 0.05 µg of the Renilla luciferase control plasmid (pRL-TK). After transfection for 48 h, luciferase activity was assessed utilizing a dual-luciferase assay kit (Promega). The relative luciferase activity was calculated by normalizing the firefly luciferase activity to the Renilla luciferase activity for each sample. Three separate experiments were conducted, and the resulting means and standard deviations were computed and reported.

The promoter region of GSDME was identified using the UCSC Genome Browser (https://genome.ucsc.edu/). The upstream and downstream sequence of 1000 base pairs (bp) relative to the transcription start site was extracted. The 2000 bp promoter sequence was systematically truncated to generate four variants: -1000 ~ 1000 bp, -1000 ~ 500 bp, -1000 ~ 0 bp, and -1000 ~ -500 bp relative to the predicted transcription start site (TSS).

### ChIP assay

The procedure was conducted according to the instructions provided with the ChIP assay kit (Cell Signaling Technology, 9005). Cells of the tested cell lines were seeded in 15 cm culture plates and allowed to grow to 70–80% confluence. To fix the cells, formaldehyde (final concentration, 1.5%) was added to the existing culture medium, and the fixation reaction was stopped by adding glycine. The cells were then collected by centrifugation, and the nuclear pellet was resuspended in ChIP Buffer. The cell lysates were sonicated, incubated overnight with 2 μg of the indicated antibodies at 4°C, and subsequently incubated overnight with protein A/G agarose beads at 4°C. The bound DNA‒protein complexes were eluted, and after a series of washes, crosslinking was reversed. The purified DNA was resuspended in TE buffer for PCR analysis. The primers used for PCR amplification were as follows: 5'-CCCCGAGGGGAAAGACTAGA-3' (forward) and 5'-CCCCACAGCACTCCTTTCAT-3' (reverse).

### siRNA transfection

The siRNA sequences were as follows:

siNC: 5'-UUCUCCGAACGUGUCACGUTT-3';

siULK1: 5'-CACTGACCTGCTCCTTAA-3';

siFUNDC1: 5'-GCAGCACCTGAAATCAACA-3';

siPARKIN#1: 5'-CGCAACAAATAGTCGGAACAT-3';

siPARKIN#2: 5'-CGTGATTTGCTTAGACTGTTT-3';

siATG5#1: 5'-CTTGTTTCACGCTATATCA-3';

siATG5#2: 5'-CCTTTCATTCAGAAGCTGTTT-3';

siATG7#1: 5'-CCCAGCTATTGGAACACTGTA-3';

siATG7#2: 5'-AGCCTCTCTATGAGTTTGA-3';

siCASP3: 5'-CCGAAAGGTGGCAACAGAATT-3';

siCASP8: 5'-GCAAUCUGUCCUUCCUGAAUUTT-3'.

All siRNAs were synthesized by GenePharma and transfected into cells using siRNA-mate (G04003-1) and Opti-MEM according to the manufacturer’s instructions.

### Immunofluorescence

Cells were seeded in plates and treated with APG-115 for 24 h. Then, the cells were incubated for 30 min at 37°C with MitoTracker Deep Red (100 nM; Invitrogen, M22426) and were then fixed with paraformaldehyde (PFA; 4%, 10 min). Next, the cells were permeabilized with 0.25% Triton X-100 (Sigma‒Aldrich, T8787) for 10 min. After three washes with PBS, the cells were stained with 4’,6-diamidino-2-phenylindole (DAPI). Confocal images were obtained using a Zeiss microscope.

### ELISA

A human IL1B/IL-1 beta ELISA kit (PI305), human IFN-γ ELISA Kit (PI521) and a human IL-18/IL-1F4 ELISA kit (PI558) were purchased from Beyotime. The concentrations of human IL1B, IFN-γ and IL-18 in the conditioned medium of cultured cells were measured in triplicate by ELISA according to the manufacturer’s protocol.

### In vivo xenograft experiments

Six-week-old female BALB/c nude mice were purchased from GemPharmatech Co.,Ltd (Guangdong, China). All animal care and experimental procedures were approved by the Institutional Animal Care and Use Committee (IACUC) of Sun Yat-sen University Cancer Center. Mice were maintained in specific pathogen-free facilities with a 12h light/dark cycle. Twenty nude mice were randomly allocated into 4 groups. A2780 cells (wild-type p53 tumor cells) in the logarithmic growth phase were digested and resuspended in a single-cell suspension, mixed with Matrigel at a Matrigel:cell ratio of 5:1, and inoculated subcutaneously into the right axilla of mice (5 × 10^6^ cells/mouse). The mice in groups 1 and 2 were inoculated with A2780 cells transfected with a nontargeting sgRNA sequence, and the mice in groups 3 and 4 were inoculated with A2780 cells transfected with a sgRNA sequence targeting the ULK1 gene. Once visible subcutaneous nodules had formed in the mice, once-daily oral treatment began. The mice in groups 1 and 3 were administered 50 mg/kg APG-115 by gavage, while the mice in groups 2 and 4 were administered an equivalent volume of solvent by gavage. During the gavage period, the long and short diameters of each subcutaneous tumor were measured and recorded every two days, and the tumor volume was calculated by the formula 0.5 × length × width^2^. When the volume of the largest subcutaneous tumor was nearly 2000 mm3, gavage was stopped, the mice were euthanized by cervical dislocation, the tumors were excised, and the tumor weights were recorded.

### Reanalysis of previously published data

The data used for pancancer analysis of MDM2 expression, gene set variation analysis (GSVA) of the mitophagy pathway, and survival analysis of patients were obtained from The Cancer Genome Atlas (TCGA, https://portal.gdc.cancer.gov/). Drug sensitivity values (IC_50_) were predicted using the “pRRophetic” package [14], and the patients were divided into dryg-sensitive and resistant groups based on the mean of the drug sensitivity values. GSVA scores were calculated using the “GSVA” package, and patient survival analysis was conducted using the “survival” package. The data used for MDM2 inhibitor sensitivity analysis and correlation analysis between TP53 mRNA expression and GSDME mRNA expression were obtained from Cancer Cell Line Encyclopedia (CCLE, https://depmap.org/portal/). The data used for ChIP analysis (GSE86164) were downloaded from Gene Expression Omnibus (GEO, https://www.ncbi. nlm.nih.gov/geo/), and the visualization of the ChIP-seq were conducted by UCSC Genome Browser (https://genome.ucsc.edu/). Comparisons between patients with wild-type p53 and patients with mutated p53 were conducted with cBioPortal (https://www.cbioportal.org/). All analyses were performed and the corresponding plots were generated in RStudio with R version 4.3.0.

### Statistical analysis

GraphPad Prism (version 8.0) and R (version 4.3.0) were used for statistical analysis. Two-tailed Student’s t test, or the chi-squared test, as appropriate, was used to assess the significance of differences. A difference was considered significant if the *P* value was < 0.05.

## Results

### High-throughput screening identified ULK1 as a synthetic lethal gene of MDM2 inhibitor

**Fig. 1:**
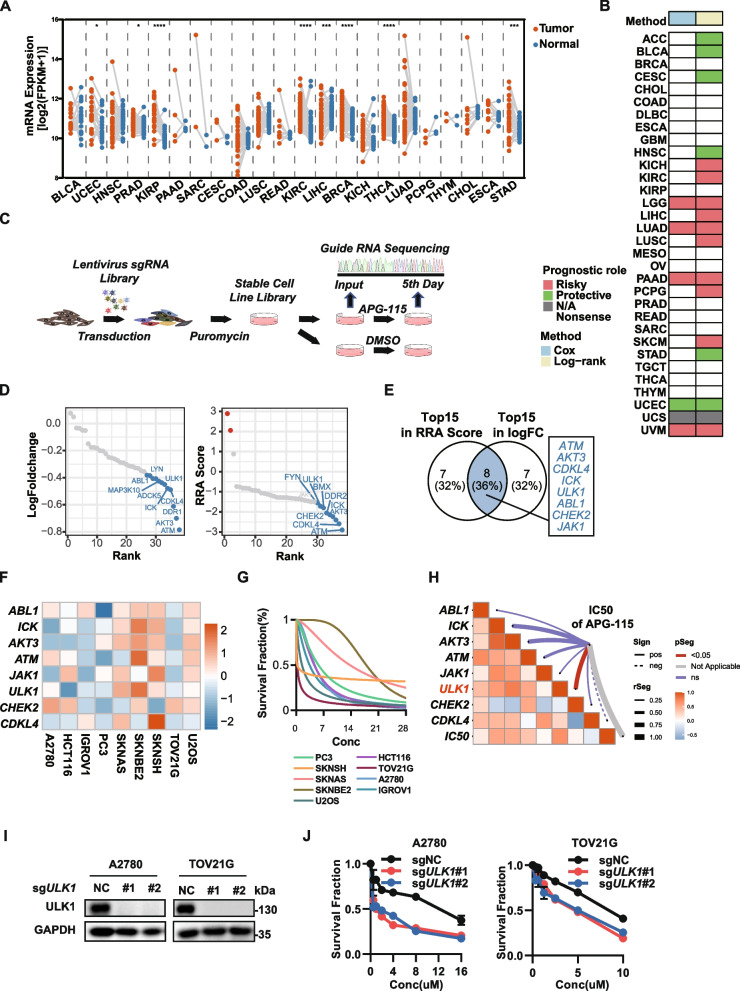
In vitro CRISPR screening identified ULK1 as a combinatorial target with an MDM2 inhibitor. **A** MDM2 mRNA expression (log2(FPKM + 1)) among all paired samples from the TCGA grouped by cancer. Each point represents one sample. The P values are based on two-tailed Student’s t test. **B** Summary of the correlation between MDM2 expression and overall survival (OS) based on univariate Cox regression and Kaplan‒Meier analyses. Red indicates that MDM2 is a risk factor affecting the prognosis of cancer patients, and green represents protective factors. Only *P* values < 0.05 are shown. **C** Schematic diagram of the in vitro screening process used to identify novel drug combinations. **D** Dot plots showing gene-specific CRISPR viability scores (log fold change and RRA scores). The points ranked in the top ten are highlighted in blue. **E** Venn diagram showing the intersection of the top 15 genes ranked by the log fold change score and the top 15 genes ranked by the RRA score. **F** Heatmap of RNA-seq analysis of nine TP53 wild-type cancer cell lines. **G** Survival curves of APG-115 in nine TP53 wild-type cancer cell lines. **H** Correlation analysis between gene expression and the IC50 of APG-115. **I** Western blot showing ULK1 protein levels in A2780 cells and TOV21-G cells expressing sgRNAs targeting ULK1. **J** Cell viability was measured by an MTT assay. A2780 cells and TOV21-G cells expressing sgRNAs targeting ULK1 were treated with APG-115 for 72 h

Approximately 50% of human cancers retain the wild-type p53 gene. However, its tumor-suppressive function is compromised by MDM2, an E3 ubiquitin ligase that targets p53 for proteasomal degradation [15]. Our analysis using the TCGA public database revealed that MDM2 is highly expressed in most tumor tissues and that its expression is negatively correlated with patient prognosis (Fig. 1A-B, Fig. S1A). Therefore, targeting MDM2 to reactivate the p53 function is considered a promising cancer therapeutic strategy, and several MDM2 inhibitors (MDM2i) have entered clinical trials. Unfortunately, recent clinical trials have shown that MDM2i perform poorly in many patients, and there is an urgent need to develop new combination therapies to guide their clinical application. To this end, we performed a high-throughput kinase CRISPR library screen and found that individual knockout of eight kinases (ATM, AKT3, CDKL4, ICK, ULK1, ABL1, CHEK2, and JAK1) markedly increased cellular sensitivity to the MDM2 inhibitor APG-115 (Fig. 1C-E). Next, to verify the screen results, we analyzed mRNA expression of these eight candidates, and also determined the half-maximal inhibitory concentrations value of APG-115 in nine kinds of p53 wild-type cancer cells (Fig. 1F-G). Correlation analysis revealed that ULK1 expression was significantly correlated with IC50 values and had the highest Pearson correlation coefficient (Fig. 1H). Furthermore, ULK1 was also the least correlated with the p53 pathway among these eight candidates (Fig. S1B-E). These results suggested that ULK1 may be the favorable co-target. ULK1, a serine/threonine kinase, is essential for initiating autophagy. It regulates the distribution and utilization of cellular resources in response to metabolic demands and is closely associated with various pathophysiological processes. ULK1 plays a crucial role in cancer, neurodegenerative diseases and so on. To verify the above screening results, we generated ULK1 knockout cell lines by constructing single guide RNA (sgRNA) sequences targeting ULK1 and selecting monoclones. Knockout of ULK1 significantly increased the sensitivity of cancer cells to APG-115, as determined by MTT and flow cytometry assays (Fig. 1I-J). Among the core protein kinases in the autophagy pathway, ULK1 plays a crucial role. However, its impact on tumor proliferation and progression is unclear. Kim et al. argue that ULK1 inhibits mTORC1's kinase activity and cell proliferation in cancers, while Yang et al. contend that ULK1 promotes epithelial ovarian cancer proliferation. Our results show that ULK1 knockdown with siRNA slightly enhances tumor cell proliferation in colony formation and cell proliferation assays (Fig. S1F-G). Conversely, ULK1 knockout using sgRNA showed no significant impact in growth rate (Fig. S1H). Therefore, ULK1 seems to have a minimal impact on tumor cell proliferation. Taken together, these results suggested that ULK1 deficiency may be synthetic lethal with MDM2i.

### ULK1 deficiency sensitizes cancer cells to MDM2i in vitro and in vivo

**Fig. 2:**
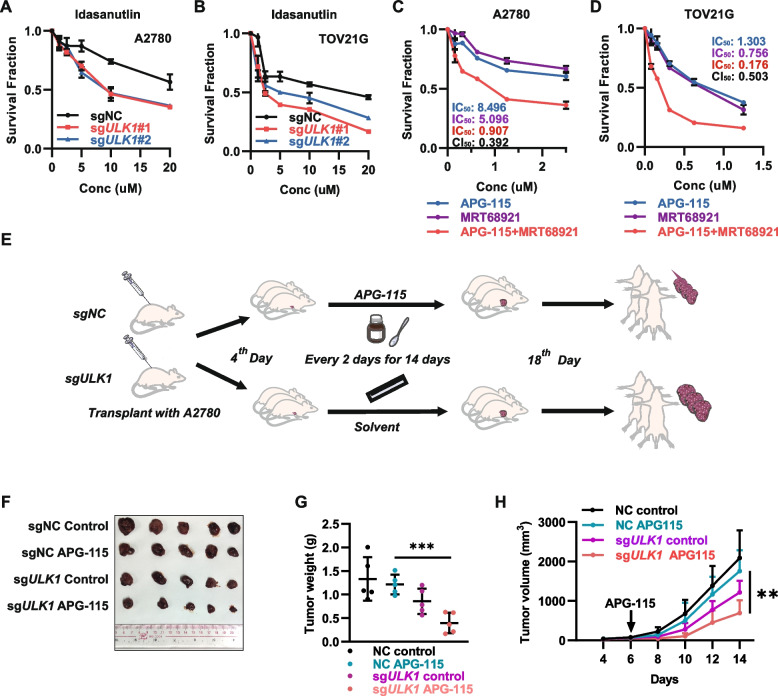
ULK1 deficiency sensitizes cancer cells to APG-115 in vitro and in vivo. **A, B** Cell viability was measured by an MTT assay. A2780 cells and TOV21-G cells expressing sgRNAs targeting ULK1 were treated with idasanutlin for 3 days. **C, D** Cell viability was measured by an MTT assay. A2780 cells or TOV21-G cells were treated with idasanutlin and an ULK1 inhibitor for 3 days. **E** Illustration showing the workflow of the animal experiments. **F** The image shows tumors excised at the end of the experiment. **G** Average tumor weight in each group of nude mice (*** *P* < 0.001). **H** Tumor volume curves of each group of nude mice (** *P* < 0.01)

To further confirm that ULK1 deficiency synergizes with p53 activation to induce synthetic lethality in cells and to determine whether this phenomenon extends beyond APG-115, we administered idasanutlin, an alternative MDM2 inhibitor, to two p53 wild-type cancer cell lines. Similar to APG-115, the depletion of ULK1 notably enchanced the susceptibility of cancer cells to idasanutlin. Additionally, we explored the potential collaborative effects between MDM2i and ULK1-specific inhibitor MRT68921, a potent specific inhibitor of ULK1 complex kinases ULK1/2. Our results reveal that combining APG-115 and MRT68921 significantly enhances cell death compared to either agent alone. The combination index values indicate a high level of synergy (Fig. 2C-D). To further investigate the effect of ULK1 knockout on the effect of MDM2i in vivo, we subcutaneously implanted tumor cells with ULK1 knockout in nude mice and began treating the mice with APG-115 every two days from day 4 after cell implantation (Fig. 2E). We measured the tumor volume periodically and measured the weight of the tumors on day 14. We found that ULK1 knockout tumors were more sensitive to APG-115 than ULK1 wild-type tumors, with a more significant reduction in tumor volume and a lower weight (Fig. 2F-H). These results suggest that targeting ULK1 increases the sensitivity of p53 wild-type tumors to the MDM2i both in vitro and in vivo.

### P53 activation combined with ULK1 deficiency can initiate pyroptosis

**Fig. 3:**
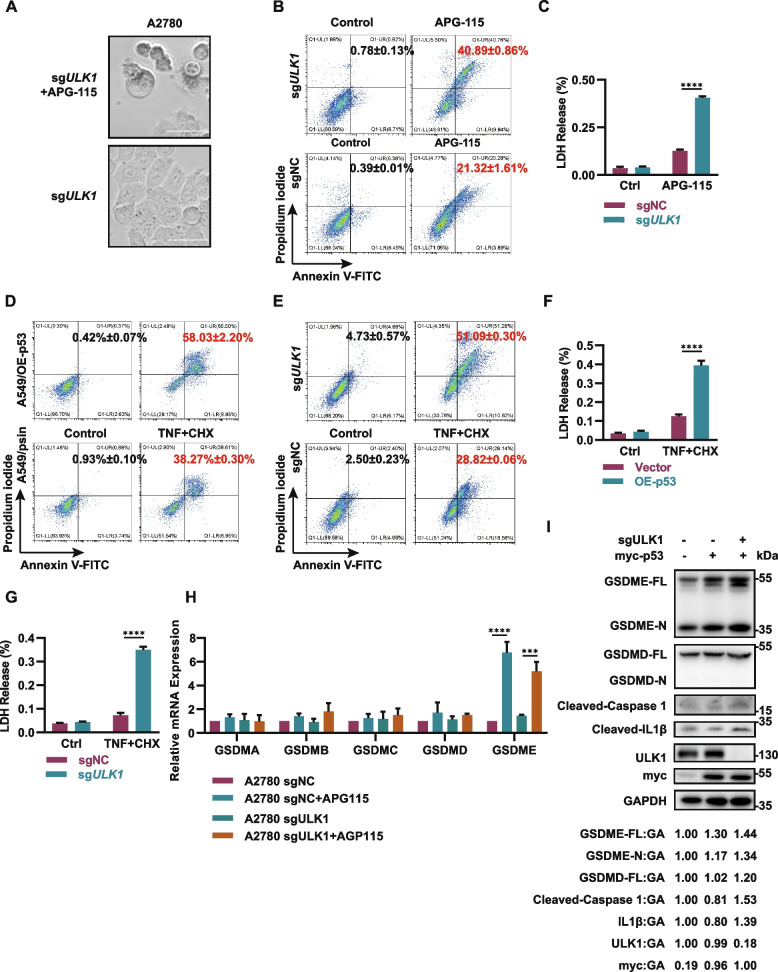
P53 activation combined with ULK1 deficiency can initiate pyroptosis. **A** Evaluation of APG-115-induced pyroptosis in A2780 sgAAV1 and A2780 sgULK1 cells by phase contrast imaging. **B** Flow cytometric analysis of Annexin V-FITC and PI staining in A2780 ULK1 knockout cells following treatment with 10 µM APG-115 for 24 h. **C** LDH release was detected using an LDH Cytotoxicity Detection Kit (Beyotime) in A2780 ULK1 knockout cells following treatment with 10 µM APG-115 for 24 h. **** *P* < 0.0001. **D, F** Flow cytometric analysis of FITC staining, PI staining, and LDH release in A549 p53-overexpressing cells following treatment with TNFα + CHX for 24 h. **** *P* < 0.0001. **E, G** Flow cytometric analysis of FITC staining, PI staining, and LDH release in A2780 ULK1 knockout cells following treatment with TNFα + CHX for 24 h. **H** RNA was extracted from the indicated cells, and the expression of GSDMA-E was analyzed by qRT‒PCR. * *P* < 0.05, *** *P* < 0.001, **** *P* < 0.0001. **I** Western blot showing GSDME protein levels in indicated A2780 cells

We further investigated why the combination of an MDM2i treatment and ULK1 deficiency effectively kills cancer cells. We pretreated ULK1 knockout cells with APG-115 for 24 h, and found that the dying cells showed evident pyroptotic morphology, with vacuole-like structures emerging from the plasma membrane and cytoplasmic contents consolidating at one pole of the cells (Fig. 3A). Furthermore, we examined the proportion of Annexin V/propidium iodide (PI) double-positive cells by flow cytometry and found that the proportion of double-positive cells increased significantly and rapidly in ULK1 knockout cells treated with MDM2i; this effect differed significantly from the observation in apoptotic cells that were initially positive for only Annexin V and then gradually became positive for PI, which suggest that the permeability of the cell membrane underwent rapid changes under this condition.(Fig. 3B). Besides, we conducted a lactate dehydrogenase (LDH) assay and observed a rapid release of intracellular LDH upon ULK1 knockout cells treated with APG-115 (Fig. 3C). These results suggest that the combination of MDM2 inhibition and ULK1 deficiency leads to a robust induction of pyroptosis in cancer cells, with plasma membrane pore formation and cell swelling. To clarify the distinct roles of p53 activation and ULK1 deficiency in inducing pyroptosis, we overexpressed p53 or knocked out ULK1 in cancer cells and treated them with TNFα + cycloheximide (CHX) to induce classical pyroptosis. Interestingly, cells showed increased susceptibility to pyroptosis upon p53 overexpression or ULK1 knockout separately, albeit to a lesser extent compared to the combined effect of p53 activation and ULK1 deficiency (Fig. 3D-G, Fig. S2A-B). The occurrence of pyroptosis is mediated by a class of GSDM family proteins that form homo-oligomers and create pores on the cell membrane, thereby inducing lytic cell death. Hence, to unveil the potential mechanism of the synthetic lethality, we examined the mRNA expression of GSDMs in cells following MDM2i/ULK1 depletion. Our findings revealed that p53 activation by APG-115 significantly increased the expression level of GSDME exclusively, with no similar effect observed for any other GSDM, while ULK1 depletion appeared to have no impact on the expression of any GSDMs (Fig. 3H, Fig. S2C). Moreover, our western blot analysis revealed ULK1 depletion enhanced GSDME cleavage, while the cleavage of GSDMD did not show significant activation (Fig. 3I). Collectively, these results suggests that p53 and ULK1 can regulate pyroptosis through different mechanisms involving GSDME. Specifically, p53 may promote the transcription of GSDME mRNA, while ULK deficiency may enhance the cleavage and activation of the GSDME protein, leading to the induction of pyroptosis respectively.

### P53 restoration induces pyroptosis by activating the transcription of GSDME directly

**Fig. 4:**
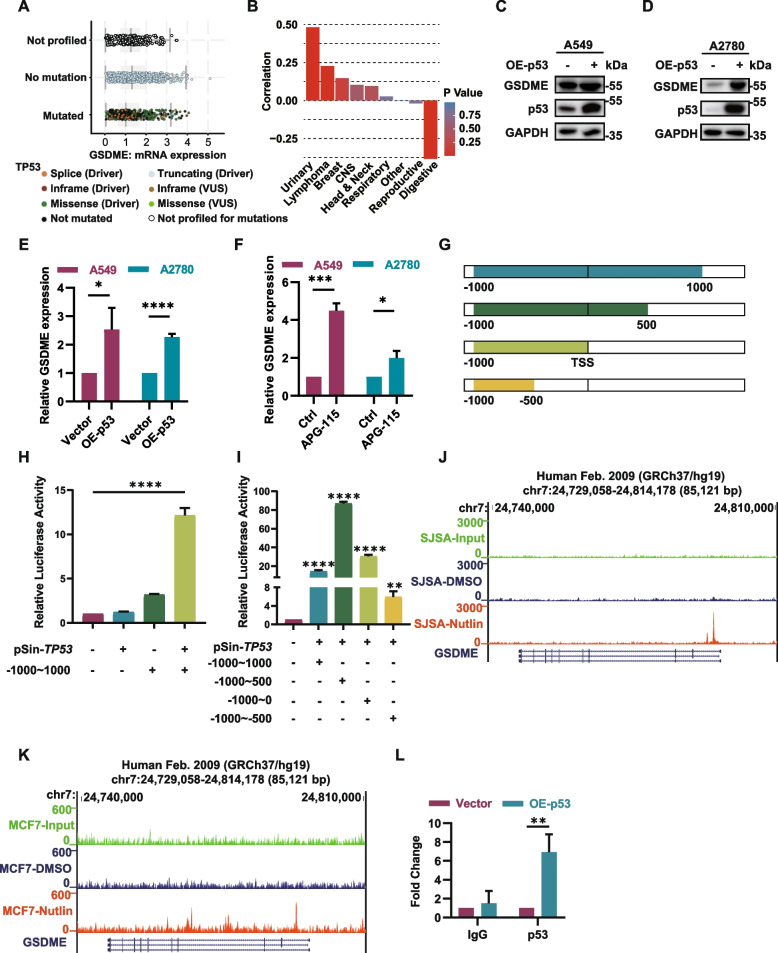
P53 directly activates the transcription of GSDME. **A** Dot plot showing the differences in the mRNA expression of GSDME between patients with different TP53 mutation statuses. Each point represents one sample. **B** Bar plot showing the correlation between the mRNA expression of GSDME and that of TP53 in 1210 cell lines from the CCLE database grouped by organ system. **C, D** p53 affects the expression of GSDME. A2780 and A549 cells were transfected with the indicated plasmids, and the expression of GSDME was determined by immunoblotting. **E, F** RNA was extracted from the indicated cells, and the expression of GSDME was analyzed by qRT‒PCR. * *P* < 0.05, *** *P* < 0.001, **** *P* < 0.0001. **G** Illustration of the truncation fragments of the GSDME promoter. **H, I** Firefly luciferase activity was measured and normalized to that of Renilla luciferase as the internal control. ** *P* < 0.01, *** *P* < 0.001, **** *P* < 0.0001. **J, K** The published ChIP-seq dataset was reanalyzed via the UCSC Genome Browser. After p53 was activated with MDM2 inhibitors, a peak appeared in the GSDME promoter region. **L** The indicated HEK-293 T p53-overexpressing cells were subjected to a ChIP assay using an antibody against p53. Isotype-matched IgG was used as a negative control. ** *P* < 0.01. The data are representative of three independent experiments

Subsequently, we delved into exploring the distinct mechanisms of p53 reactivation and ULK1 deficiency in GSDME mediated pyroptosis activation, separately. Initially, to further investigate and validate the molecular mechanisms of p53 functional restoration in GSDME expression, we analyzed the relationships between the p53 mutation status and the mRNA expression levels of GSDMs using the cBioPortal database. In patients with p53-mutant tumors, the mRNA expression level of GSDME was significantly lower than that in patients with wild-type tumors, whereas other GSDM family genes did not exhibit this pattern. This further confirmed that p53 exclusively regulated pyroptosis by controlling GSDME expression (Fig. 4A, Fig. S2D-G). Through analysis of CCLE data from 1210 cell lines across multiple cancers, we found that TP53 expression was positively correlated with the mRNA level of GSDME in the vast majority of cancers (Fig. 4B). Western blot and quantitative PCR (qPCR) analyses also showed that the expression level of GSDME was significantly upregulated following p53 overexpression or activation by APG-115 (Fig. 4C-F). These results suggested that p53 played a crucial role in the transcription of GSDME. To determine whether p53 was directly involved in the transcriptional activation of GSDME, we conducted a dual-luciferase reporter assay initially. As expected, significant binding of p53 to the promoter region of GSDME was observed. Further sequence truncation identified the primary region of p53 binding to be located between 1- 500 base pairs downstream of the transcription start site. (Fig. 4G-I). Furthermore, the analysis of publicly available chromatin immunoprecipitation–sequencing (ChIP-seq) datasets revealed a significant increase in p53 binding near the GSDME promoter region upon upregulation of p53 activation by treatment with the MDM2 inhibitor nutlin (Fig. 4J-K). To validate this result, we also performed ChIP‒qPCR analysis and observed a significant increase in p53 protein enrichment at the GSDME promoter region upon p53 overexpression (Fig. 4L). These results suggested that p53 directly mediated the transcriptional activation of GSDME, which induced basal level of pyroptosis.

### ULK1 deficiency induces GSDME cleavage activation and pyroptosis through upregulation of ROS

**Fig. 5:**
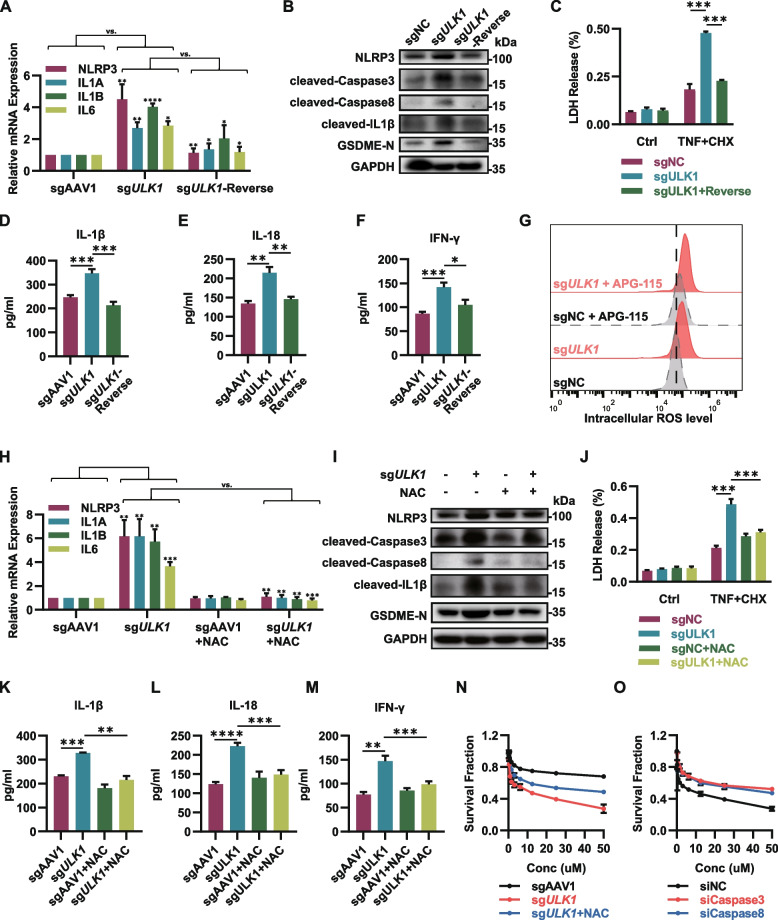
ULK1 deficiency induces GSDME activation through upregulation of ROS. **A** RNA was extracted from the indicated cells, and the expression of NLRP3, caspase 8 and IL1B was analyzed by qRT‒PCR. **B** Immunoblot analysis of NLRP3-Caspase pathway proteins and GAPDH from extracts of the indicated cells. **C** LDH release was detected using an LDH Cytotoxicity Detection Kit (Beyotime) in indicated cells. **D, E** The indicated A2780 cells were treated with 10 µM APG-115 for 24 h. IL-1β, IL-18 and IFN-γ concentrations in the indicated culture media were measured by ELISA. **G** Quantification of intracellular ROS in the indicated cells following treatment with 10 µM APG-115 for 24 h. **H** The indicated A2780 cells were treated with 4 mM NAC for 24 h. RNA was extracted from the indicated cells, and the expression of NLRP3, caspase 8 and IL1B was analyzed by qRT‒PCR. **I** The indicated A2780 cells were treated with 4 mM NAC for 24 h. Immunoblot analysis of NLRP3-Caspase pathway proteins and GAPDH from extracts of the indicated cells. **J** LDH release was detected using an LDH Cytotoxicity Detection Kit (Beyotime) in indicated cells. **K-M** The indicated A2780 cells were treated with 10 µM APG-115 and 4 mM NAC for 24 h. IL-1β, IL-18 and IFN-γ concentrations in the indicated culture media were measured by ELISA. **N** Survival fractions of the indicated cells following treatment with APG-115 and NAC for 72 h. **O** Survival fractions of control, siCaspase3 and siCaspase8 cells following treatment with APG-115

Prior researches have shown that pyroptosis primarily occurs in macrophages and is mediated by inflammatory caspases that activate GSDMD. However, researchers have also found that in epithelial-derived cells, especially in tumor cells, the induction of pyroptosis is mainly mediated by GSDME, which is activated by caspase-3/8 and other apoptosis-related caspases. To delve deeper into how GSDME cleavage is promoted in ULK1-deficient cancer cells, we performed qPCR and western blot analysis. Our results unveiled a significant upregulation of cleaved forms of caspase-3 and caspase-8. Notably, the expression level of NLRP3 and the release of IL-1β, IL-18, IFN-γ and LDH were also elevated in response to ULK1 depletion. And these effects were reversed upon the restoration of wild-type ULK1 (Fig. 5A-F). All these results suggested that NLRP3-caspase signaling axis was activated in ULK1 deficiency cancer cells. Unlike immune cells, it has been reported that epithelial-derived cells can also activate non-inflammatory caspases through ROS-NLRP3 axis. As expected, measurement of overall intracellular ROS levels confirmed a significant increase in cancer cells following ULK1 depletion (Fig. 5G). To further validated these findings, we used ROS scavenger N-acetylcysteine (NAC) to inhibit ROS level, and our results showed that treatment with NAC significantly reversed the cleavage of caspase-3/8 and the activation of GSDME. Additionally, it also significantly blocked the expression and release of pyroptosis-related inflammatory factors (Fig. 5H-M). Furthermore, NAC treatment or siRNA-mediated knockdown of Caspase-3 and Caspase-8 significantly reduced the synergistic cytotoxic effect of ULK1 deficiency and the APG-115 (Fig. 5N-O). These results collectively suggest that ULK1 deficiency can further promote pyroptosis by elevated ROS level, activate of the NLRP3-caspase signaling axis and promote the cleavage of GSDME.

### The synergistic induction of pyroptosis by p53 activation and ULK1 depletion is critically dependent on mitochondria quality control

**Fig. 6:**
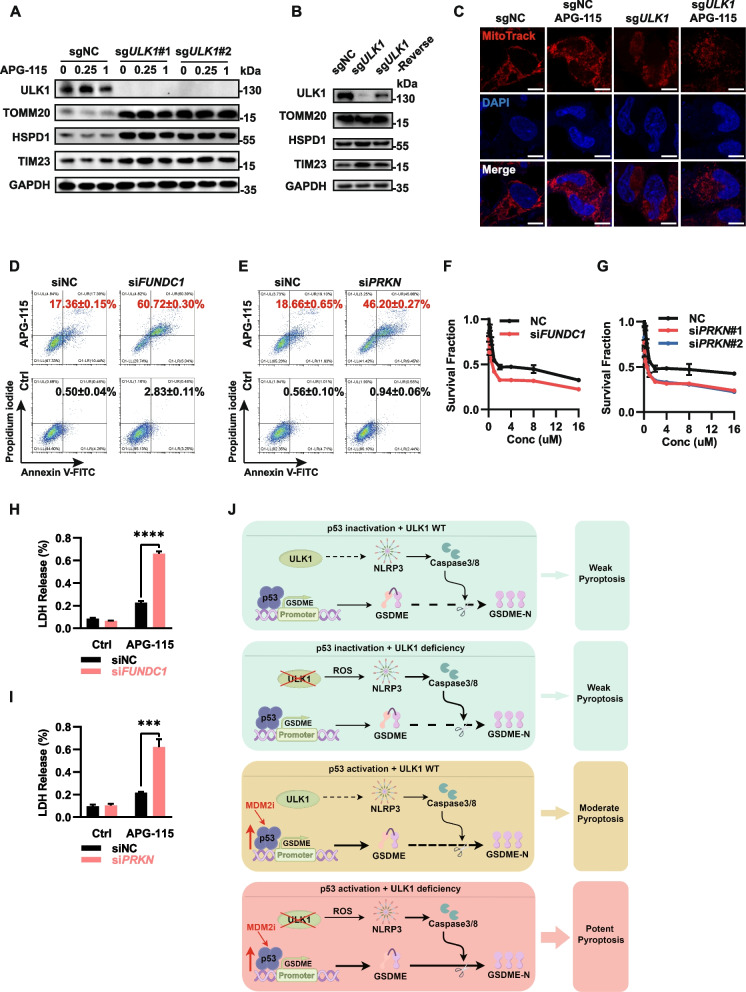
The synergistic induction of pyroptosis by p53 activation and ULK1 depletion depends on mitochondria quality control. **A, B** Immunoblot analysis of ULK1, TOMM20, HSPD1, TIM23 and GAPDH expression in indicated cells. The indicated A2780 cells were treated with10 µM APG-115 for 24 h. **C** Immunofluorescence images of A2780 cells with deletion of ULK1 and treatment with 10 µM APG-115 for 24 h. The cells were costained with MitoTracker Deep Red and DAPI. The white line represents 10 µm. **D, E** Flow cytometric analysis of FITC- and PI-stained control, siFUNDC1, and siPARK A2780 cells treated with 10 µM APG-115 for 24 h. **F, G** Survival fractions of control, siFUNDC1, and siPARK A2780 cells following treatment with 10 µM APG-115 for 24 h. **H, I** LDH release was detected in models of mitophagy deficiency or macroautophagy deficiency following treatment with 10 µM APG-115 for 24 h. ns: *P* > 0.05, ** *P* < 0.01, *** *P* < 0.001, **** *P* < 0.0001. **J** Schematic of mechanisms underlying the synergy between MDM2i and ULK1 deficiency in TP53 wild-type cells. See main text for a detailed description

As an autophagy regulator with kinase activity, ULK1 can directly regulate mitochondrial quality control to affect the overall redox balance in the cell. When ULK1 is deficient, damaged mitochondria accumulate, leading to increased ROS release, which may be a key mechanism underlying the activation of the NLRP3-caspase signaling axis and GSDME dependent pyroptosis. To validate this hypothesis, we conducted western blotting and observed a significant increase in the intracellular expression levels of TOMM20, HSPD1 and TIM23 (proteins localized in mitochondria) upon ULK1 knockout (Fig. 6A-B). Additionally, labeling of intracellular mitochondria with MitoTracker Deep Red revealed punctate accumulation of mitochondria upon ULK1 knockout, indicating blockade of mitophagy and accumulation of mitochondrial damage (Fig. 6C). Furthermore, we performed siRNA-mediated knockdown of FUNDC1 and Parkin to establish a model of mitophagy deficiency. And subsequent assessment via MTT assays and flow cytometry revealed that the synergistic effect of ULK1 knockout via APG-115 treatment was effectively replicated in the model of mitophagy deficiency (Fig. 6F-I). We also observed a significant increase in expression of NLRP3, active caspase-8, cleaved caspase-3, and LDH release upon knocking down key mitophagy genes, akin to the effect observed following ULK1 knockout (Fig. S3A-H). All these findings indicated that the key mechanism underlying the synergistic lethality of ULK1 deficiency and MDM2 inhibitor-mediated p53 activation is the regulation of mitochondrial quality control. The deficiency of key factors involved in mitophagy can also synergize with p53 activation to induce pyroptosis.

In summary, our study demonstrates that targeting ULK1 in combination with p53 reactivation can induce synthetic lethality in cells by promoting pyroptosis. Firstly, since pyroptosis and apoptosis share upstream caspases, the key to whether a cell undergoes pyroptosis lies in the expression of GSDM family proteins. We found that p53 can directly induce the transcriptional activation of GSDME, leading to a switch from apoptosis to pyroptosis. However, the degree of caspase activation is low during this process, the cellular response to MDM2 inhibitors is insufficient. Secondly, in ULK1-deficient cells, the autophagic degradation process is impaired, leading to dysregulation of mitochondrial quality control and accumulation of damaged mitochondria. This results in increased ROS production and activation of NLRP3, which in turn induces the cleavage and activation of a series of death-related caspases. However, in cancer cells, the expression of GSDM family genes is often downregulated for various reasons, making it insufficient to activate the pyroptosis signaling pathway. Finally, in ULK1-deficient cells, the use of MDM2 inhibitors can upregulate the expression of GSDME and simultaneously activate caspase signaling to induce its cleavage and activation, thereby amplifying the pyroptosis signal and producing a synthetic lethal effect.

### Mitophagy deficiency combined with p53 activation can be utilized to reverse platinum resistance

**Fig. 7:**
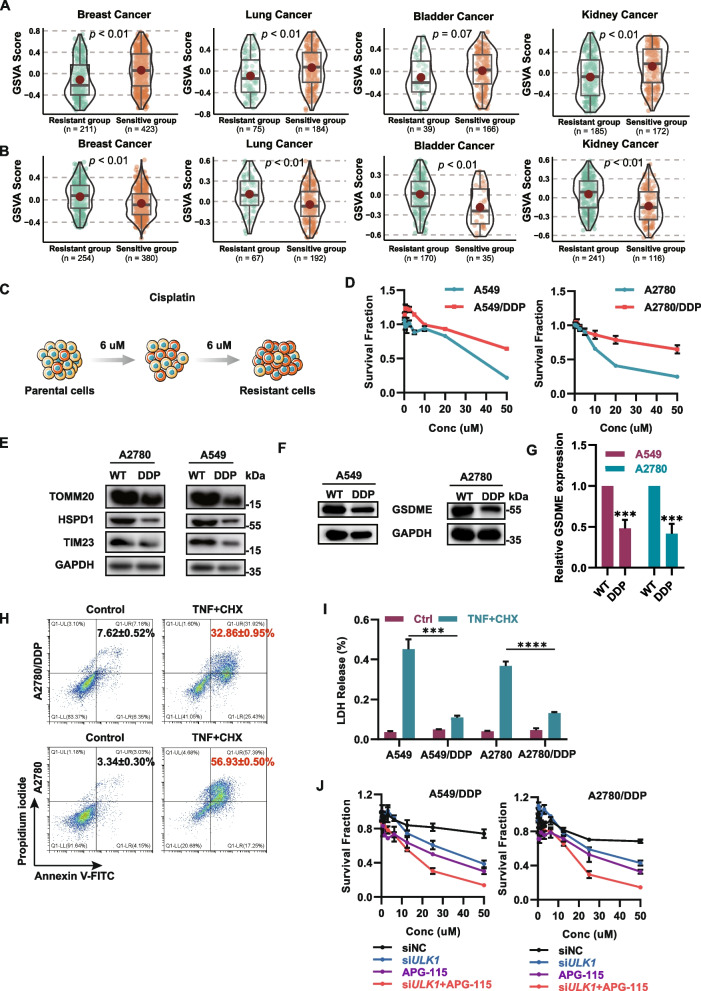
Combined targeting of mitophagy and activation of p53 could be used to reverse platinum resistance. **A** GSVA scores of mitophagy-related genes in cisplatin-sensitive and cisplatin-resistant samples of four tumors from TCGA. The sensitivity of the tumor samples was calculated using the "pRRophetic" R package, and the optimal cutoff value of the ROC curve was selected as the cutoff point between sensitivity and resistance based on the Youden index. The P values were calculated using the Wilcoxon rank–sum test. **B** GSVA scores of pyroptosis-related genes in cisplatin-sensitive and cisplatin-resistant samples of four tumors from TCGA. **C** Illustration showing the process for generating cisplatin-resistant cell lines. **D** Survival fractions of the indicated cisplatin-resistant cells following treatment with cisplatin for 72 h. **E** Immunoblotting of ULK1, TOMM20, HSPD1, TIM23 and GAPDH in extracts of cisplatin-resistant cells. **F, G** GSDME expression in cisplatin-resistant cells. *** *P* < 0.001. **H, I** The indicated cisplatin-resistant cells were treated with TNFα + CHX for 24 h. ** *P* < 0.01, *** *P* < 0.001, **** *P* < 0.0001. **J** Survival fractions of the indicated cisplatin-resistant cells following treatment with cisplatin for 72 h

Platinum is commonly utilized as the primary agent for the treatment of solid tumors, and resistance to this drug is a major contributing factor to tumor recurrence and metastasis. Mitophagy has been identified as a critical mechanism in this process. We previously performed gene set variation analysis (GSVA) to evaluate the enrichment of the pro-mitophagy and pyroptosis pathways in cisplatin-sensitive and cisplatin-resistant patients with wild-type p53. The results demonstrated significant upregulation of pro-mitophagy gene set activity and downregulation of pyroptosis pathway activity in cisplatin-resistant patient samples compared to cisplatin-sensitive patient samples, as expected (Fig. 7A-B). To investigate the mechanism of cisplatin resistance and potential therapeutic strategies to overcome it, two p53 wild-type cell lines, A549 and A2780, were treated with sublethal concentrations of cisplatin for prolonged periods, resulting in the generation of cisplatin-resistant cell lines (A549/DDP and A2780/DDP). The degree of resistance was subsequently confirmed through an MTT assay (Fig. 7C-D). Subsequently, the cisplatin-resistant cell lines were found to exhibit elevated expression levels of the key gene associated with mitophagy and a notable decrease in the expression of GSMDE, the key gene of pyroptosis, as determined through western blot analysis (Fig. 7E-G). Furthermore, following treatment with TNFα + CHX for 24 h, a marked reduction in lactate dehydrogenase release, accompanied by a significant decrease in Annexin V/PI double-positive signals, was observed in cisplatin-resistant cells compared to cisplatin-sensitive cells (Fig. 7H-I). These findings indicate that in cisplatin-resistant cell lines, mitophagy is increased, while pyroptosis is significantly inhibited. Consequently, it is plausible to hypothesize that a combination therapy targeting mitophagy and p53-mediated activation of pyroptosis could reverse cisplatin resistance. To confirm this hypothesis, drug combination experiments were conducted using the MTT assay, revealing that ULK1 knockdown combined with APG-115 treatment effectively induced the death of cisplatin-resistant cells (Fig. 7J). This finding indicates that the activation of mitophagy and decrease in GSDME expression may play a crucial role in conferring cisplatin resistance on tumor cells. Moreover, concurrent treatment with a ULK1 inhibitor and activation of p53 has shown promise in effectively eliminating drug-resistant cells, providing a theoretical foundation for potential clinical interventions in cisplatin-refractory patients.

## Discussion

**Fig. 8:**
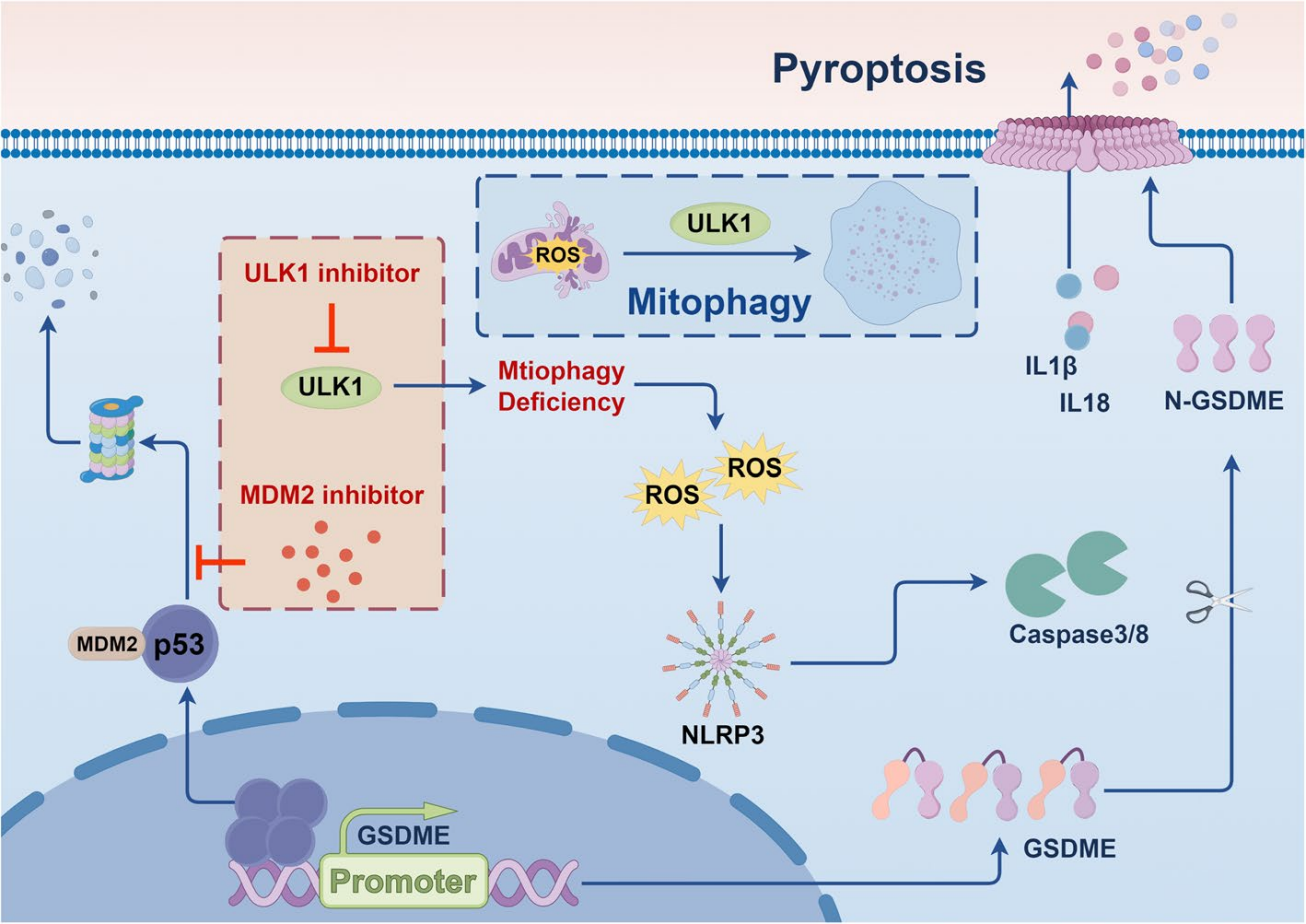
Schematic of ULK1 deficiency and p53 activation promote pyroptosis by activating GSDME directly (By Figdraw)

MDM2-mediated degradation of p53 is a key reason for its functional deficiency in the vast majority of tumors. Inhibiting MDM2 to restore p53 function is promising but has shown limited clinical efficacy, requiring better therapeutic indications and combination strategies. Our previous high-throughput screening results indicated that depletion of ULK1 significantly increased the sensitivity of tumor cells to MDM2 inhibitors and induced classical pyroptosis in these cells. This phenomenon can be attributed to the direct recognition of the GSDME gene promoter region by P53, which leads to transcriptional activation of GSDME expression and initiation of pyroptosis. Additionally, ULK1-deficient cells exhibit mitophagy defects and ROS accumulation, which in turn activate the NLRP3-Caspase8 signaling pathway and increase GSDME cleavage, thereby amplifying the pyroptotic signaling cascade (Fig. 8). In addition, we found that mitophagy was overactivated in platinum-resistant cells and that targeting ULK1 in combination with MDM2 inhibitor treatment effectively induced pyroptosis and reversed platinum resistance.

Pyroptosis is a distinct form of programmed cell death that has been linked to chemoresistance and immunotherapy failure. Its regulatory mechanisms are not well understood, which hinders the clinical implementation of pyroptosis-inducing strategies for tumor therapy. The primary effector molecules in pyroptosis are a subset of GSDM family proteins, which can form homopolymers and disrupt membrane permeability, leading to cellular disintegration and death [4, 16, 17].. Akino K. et al. discovered that GSDME is typically not expressed in tumor cells [6, 18], potentially because of hypermethylations in the promoter region, which inhibits its transcription. Furthermore, research conducted by Zhao et al. revealed that GSDME-N exhibits increased instability and susceptibility to ubiquitination and subsequent degradation through the proteasome pathway [9], ultimately leading to chemoresistance in tumors. Our research has shown that p53 can directly bind to the promoter region of GSDME and activate its transcription, thereby inducing pyroptosis. This finding not only provides further evidence for the role of p53 as a central regulator of cell fate but also offers theoretical support for the application of p53 functional restoration therapy.

In 2004, Vassilev identified and documented the initial small molecule inhibitor of the MDM2 protein, known as Nutlin [19]. Over the subsequent decade, the advancement of MDM2 inhibitors has attracted considerable attention, resulting in the progression of certain inhibitors to clinical trials [20]. Although small molecule inhibitors targeting the MDM2 protein, such as APG-115, have clinical potential, their use is fraught with various challenges [21–27]. The negative feedback loop between p53 and MDM2 protein expression may limit the efficacy of small molecule MDM2 inhibitors, and widespread cellular apoptosis in normal tissues may cause toxicity. Several small molecule inhibitors targeting MDM2 have been terminated due to inadequate clinical performance, and resistance to MDM2 inhibitors may arise from elevated MDMX protein levels and p53 mutations [2, 3, 20, 23, 28–32]. It has been reported that the combined use of BCL2, CDK4/6 inhibitors or MEK inhibitors can synergistically enhance the anti-tumor effects of MDM2 inhibitors in different types of tumors. The underlying mechanisms involve either inducing cell cycle arrest or reactivating apoptotic signals [33–35]. Inhibitors targeting MDM2 alone have limited efficacy, and researchers are seeking to find dual-target inhibitors through various approaches such as high-throughput drug screening and structural optimization. For instance, dual-target inhibitors targeting MDM4/MDMX and XIAP have shown good tumor inhibition potential and avoidance of MDM2 feedback activation in both in vitro and in vivo models [36–38]. Indeed, our high-throughput CRISPR screen showed that knockout of the ULK1 gene increased sensitivity to APG-115 in wild-type p53 tumors. Downregulation of ULK1 may trigger pyroptosis by affecting mitochondrial integrity, increasing intracellular ROS levels, and activating Caspase family proteins via NLRP3 signaling, leading to GSDME cleavage-mediated activation. Additionally, combining APG-115 with an ULK1 inhibitor reduced MDM2 negative feedback and increased p53 activation, suggesting potential clinical benefits.

## Conclusions

In conclusion, we developed an innovative approach of targeting crucial genes associated with mitophagy in conjunction with MDM2 inhibitor treatment to effectively induce tumor cell death by enhancing pyroptosis. MDM2 inhibitors increase the p53 protein level, leading to increased transcriptional activation of the pivotal pyroptosis molecule GSDME. Conversely, ULK1 deficiency-induced accumulation of ROS activates the inflammasome-Caspase axis, promoting GSDME cleavage and facilitating pyroptosis initiation. The discovery of the synergistic effect of this combination therapy and the elucidation of the underlying molecular mechanisms indicates that cancers with excessive mitophagy activation are more amenable to treatment with MDM2 inhibitors. This finding opens new avenues for antitumor therapies aimed at activating p53. Additionally, our research provides an important theoretical basis for the selection of clinical treatment strategies for cisplatin-resistant patients.

### Supplementary Information

Supplementary Material 1.

## Data Availability

The published data used in this study were obtained mainly from the following public databases: Gene Expression Omnibus (GEO, https://www.ncbi. nlm.nih.gov/geo/) (GSE86164), The Cancer Genome Atlas (TCGA, https://portal.gdc.cancer.gov/), Cancer Cell Line Encyclopedia (CCLE, https://depmap.org/portal/), and cBioPortal (http://cbioportal.org/). Other data generated in this study are available within the article and its supplementary data files.

## Abbreviations

ATG: Autophagy-related
baf A1: Bafilomycin A1
CHX: Cycloheximide
GSDM: Gasdermin
HSPD1: Heat shock protein family D (Hsp60) member 1
MDM2: MDM2 proto-oncogene
ROS: Reactive oxygen species
ULK1: Unc-51 like autophagy activating kinase 1

## References

[1] Boutelle AM, Attardi LD. p53 and Tumor Suppression: It Takes a Network. Trends Cell Biol. 2021;31:298–310. 33518400 10.1016/j.tcb.2020.12.011 PMC7954925

[2] Khoo KH, Verma CS, Lane DP. Drugging the p53 pathway: understanding the route to clinical efficacy. Nat Rev Drug Discov. 2014;13:217–36. 24577402 10.1038/nrd4236

[3] Mullard A. p53 programmes plough on. Nat Rev Drug Discov. 2020;19:497–500. 32665592 10.1038/d41573-020-00130-z

[4] Shi J, Gao W, Shao F. Pyroptosis: Gasdermin-Mediated Programmed Necrotic Cell Death. Trends Biochem Sci. 2017;42:245–54. 27932073 10.1016/j.tibs.2016.10.004

[5] Moussette S, Al Tuwaijri A, Kohan-Ghadr H-R, Elzein S, Farias R, Bérubé J, et al. Role of DNA methylation in expression control of the IKZF3-GSDMA region in human epithelial cells. PLoS ONE. 2017;12: e0172707. 28241063 10.1371/journal.pone.0172707 PMC5328393

[6] Wang Y, Gao W, Shi X, Ding J, Liu W, He H, et al. Chemotherapy drugs induce pyroptosis through caspase-3 cleavage of a gasdermin. Nature. 2017;547:99–103. 28459430 10.1038/nature22393

[7] Ibrahim J, De Schutter E, Op de Beeck K. GSDME: a potential ally in cancer detection and treatment. Trends Cancer. 2021;7:392–4. 33422423 10.1016/j.trecan.2020.12.002

[8] Wei Y, Lan B, Zheng T, Yang L, Zhang X, Cheng L, et al. GSDME-mediated pyroptosis promotes the progression and associated inflammation of atherosclerosis. Nat Commun. 2023;14:929. 36807553 10.1038/s41467-023-36614-w PMC9938904

[9] OTUD4-mediated GSDME deubiquitination enhances radiosensitivity in nasopharyngeal carcinoma by inducing pyroptosis - PubMed. https://pubmed.ncbi.nlm.nih.gov/36411454/ (accessed 3 Mar2024). 10.1186/s13046-022-02533-9 PMC9677691 36411454

[10] Okondo MC, Johnson DC, Sridharan R, Go EB, Chui AJ, Wang MS, et al. DPP8 and DPP9 inhibition induces pro-caspase-1-dependent monocyte and macrophage pyroptosis. Nat Chem Biol. 2017;13:46–53. 27820798 10.1038/nchembio.2229 PMC5477230

[11] K N, Ke W, A M, Dl D, R R, Y Z et al*.* Activity of caspase-8 determines plasticity between cell death pathways. Nature. 2019;575. 10.1038/s41586-019-1752-8. 10.1038/s41586-019-1752-8 31723262

[12] Aguilar A, Lu J, Liu L, Du D, Bernard D, McEachern D, et al. Discovery of 4-((3′ *R* ,4′ *S* ,5′ *R* )-6″-Chloro-4′-(3-chloro-2-fluorophenyl)-1′-ethyl-2″-oxodispiro[cyclohexane-1,2′-pyrrolidine-3′,3″-indoline]-5′-carboxamido)bicyclo[2.2.2]octane-1-carboxylic Acid (AA-115/APG-115): A Potent and Orally Active Murine Double Minute 2 (MDM2) Inhibitor in Clinical Development. J Med Chem. 2017;60:2819–39. 28339198 10.1021/acs.jmedchem.6b01665 PMC5394527

[13] Wang B, Wang M, Zhang W, Xiao T, Chen C-H, Wu A, et al. Integrative analysis of pooled CRISPR genetic screens using MAGeCKFlute. Nat Protoc. 2019;14:756–80. 30710114 10.1038/s41596-018-0113-7 PMC6862721

[14] Geeleher P, Cox N, Huang RS. pRRophetic: An R Package for Prediction of Clinical Chemotherapeutic Response from Tumor Gene Expression Levels. PLoS ONE. 2014;9: e107468. 25229481 10.1371/journal.pone.0107468 PMC4167990

[15] Shangary S, Wang S. Targeting the MDM2-p53 interaction for cancer therapy. Clin Cancer Res. 2008;14:5318–24. 18765522 10.1158/1078-0432.CCR-07-5136 PMC2676446

[16] Ding J, Wang K, Liu W, She Y, Sun Q, Shi J, et al. Pore-forming activity and structural autoinhibition of the gasdermin family. Nature. 2016;535:111–6. 27281216 10.1038/nature18590

[17] Broz P, Pelegrín P, Shao F. The gasdermins, a protein family executing cell death and inflammation. Nat Rev Immunol. 2020;20:143–57. 31690840 10.1038/s41577-019-0228-2

[18] Akino K, Toyota M, Suzuki H, Imai T, Maruyama R, Kusano M, et al. Identification of DFNA5 as a target of epigenetic inactivation in gastric cancer. Cancer Sci. 2007;98:88–95. 17083569 10.1111/j.1349-7006.2006.00351.x PMC11158324

[19] Vassilev LT, Vu BT, Graves B, Carvajal D, Podlaski F, Filipovic Z, et al. In vivo activation of the p53 pathway by small-molecule antagonists of MDM2. Science. 2004;303:844–8. 14704432 10.1126/science.1092472

[20] Konopleva M, Martinelli G, Daver N, Papayannidis C, Wei A, Higgins B, et al. MDM2 inhibition: an important step forward in cancer therapy. Leukemia. 2020;34:2858–74. 32651541 10.1038/s41375-020-0949-z

[21] Tolcher AW, Fang DD, Li Y, Tang Y, Ji J, Wang H, et al. A phase Ib/II study of APG-115 in combination with pembrolizumab in patients with unresectable or metastatic melanomas or advanced solid tumors. Ann Oncol. 2019;30: i2. 10.1093/annonc/mdz027

[22] Tolcher AW, Reeves JA, McKean M, Chmielowski B, Beck JT, Shaheen MF, et al. Preliminary results of a phase II study of alrizomadlin (APG-115), a novel, small-molecule MDM2 inhibitor, in combination with pembrolizumab in patients (pts) with unresectable or metastatic melanoma or advanced solid tumors that have failed immuno-oncologic (I-O) drugs. J Clin Oncol. 2021;39:2506–2506.34097441 10.1200/JCO.2021.39.15_suppl.2506

[23] Wang S, Zhao Y, Aguilar A, Bernard D, Yang C-Y. Targeting the MDM2-p53 Protein-Protein Interaction for New Cancer Therapy: Progress and Challenges. Cold Spring Harb Perspect Med. 2017;7: a026245. 28270530 10.1101/cshperspect.a026245 PMC5411684

[24] A novel small molecule inhibitor of MDM2-p53 (APG-115) enhances radiosensitivity of gastric adenocarcinoma - PubMed. https://pubmed.ncbi.nlm.nih.gov/29716622/ (accessed 3 Mar2024). 10.1186/s13046-018-0765-8 PMC5930807 29716622

[25] Dd F, Q T, Y K, T R, Q W, N L *et al.* MDM2 inhibitor APG-115 exerts potent antitumor activity and synergizes with standard-of-care agents in preclinical acute myeloid leukemia models. Cell death discovery. 2021;7. 10.1038/s41420-021-00465-5. 10.1038/s41420-021-00465-5 PMC8093284 33941774

[26] Zhang X, Wen X, Yang C, Zeng S, Men L, Wang H, et al. A phase I study of a novel MDM2-P53 antagonist APG-115 in Chinese patients with advanced soft tissue sarcomas. J Clin Oncol. 2019;37:3124–3124.31449470 10.1200/JCO.2019.37.15_suppl.3124

[27] Zhang X, Wen X, Chen G, Zeng S, Men L, Wang H, et al. Phase I study results of APG-115, a MDM2-p53 antagonist in Chinese patients with advanced liposarcoma and other solid tumors. J Clin Oncol. 2020;38:11542–11542. 10.1200/JCO.2020.38.15_suppl.11542

[28] Elucidation of Acquired Resistance to Bcl-2 and MDM2 Inhibitors in Acute Leukemia In Vitro and In Vivo - PubMed. https://pubmed.ncbi.nlm.nih.gov/25754349/ (accessed 3 Mar2024). 10.1158/1078-0432.CCR-14-2506 PMC4957562 25754349

[29] Ray-Coquard I, Blay J-Y, Italiano A, Le Cesne A, Penel N, Zhi J, et al. Effect of the MDM2 antagonist RG7112 on the P53 pathway in patients with MDM2-amplified, well-differentiated or dedifferentiated liposarcoma: an exploratory proof-of-mechanism study. Lancet Oncol. 2012;13:1133–40. 23084521 10.1016/S1470-2045(12)70474-6

[30] Andreeff M, Kelly KR, Yee K, Assouline S, Strair R, Popplewell L, et al. Results of the Phase I Trial of RG7112, a Small-Molecule MDM2 Antagonist in Leukemia. Clin Cancer Res. 2016;22:868–76. 26459177 10.1158/1078-0432.CCR-15-0481 PMC4809642

[31] Wade M, Wong ET, Tang M, Stommel JM, Wahl GM. Hdmx modulates the outcome of p53 activation in human tumor cells. J Biol Chem. 2006;281:33036–44. 16905769 10.1074/jbc.M605405200

[32] Garcia D, Warr MR, Martins CP, Brown Swigart L, Passegué E, Evan GI. Validation of MdmX as a therapeutic target for reactivating p53 in tumors. Genes Dev. 2011;25:1746–57. 21852537 10.1101/gad.16722111 PMC3165938

[33] Vilgelm AE, Saleh N, Shattuck-Brandt R, Riemenschneider K, Slesur L, Chen SC. MDM2 antagonists overcome intrinsic resistance to CDK4/6 inhibition by inducing p21. Sci Transl Med. 2019;11:eaav7171. 31413145 10.1126/scitranslmed.aav7171 PMC7584132

[34] Pan R, Ruvolo V, Mu H, Leverson JD, Nichols G, Reed JC, et al. Synthetic Lethality of Combined Bcl-2 Inhibition and p53 Activation in AML: Mechanisms and Superior Antileukemic Efficacy. Cancer Cell. 2017;32:748–760.e6. 29232553 10.1016/j.ccell.2017.11.003 PMC5730338

[35] Berberich A, Kessler T, Thomé CM, Pusch S, Hielscher T, Sahm F, et al. Targeting Resistance against the MDM2 Inhibitor RG7388 in Glioblastoma Cells by the MEK Inhibitor Trametinib. Clin Cancer Res. 2019;25:253–65. 30274984 10.1158/1078-0432.CCR-18-1580

[36] Gu L, Zhang H, Liu T, Zhou S, Du Y, Xiong J, et al. Discovery of Dual Inhibitors of MDM2 and XIAP for Cancer Treatment. Cancer Cell. 2016;30:623–36. 27666947 10.1016/j.ccell.2016.08.015 PMC5079537

[37] Park DE, Cheng J, Berrios C, Montero J, Cortés-Cros M, Ferretti S, et al. Dual inhibition of MDM2 and MDM4 in virus-positive Merkel cell carcinoma enhances the p53 response. Proc Natl Acad Sci USA. 2019;116:1027–32. 30598450 10.1073/pnas.1818798116 PMC6338866

[38] Chang YS, Graves B, Guerlavais V, Tovar C, Packman K, To K-H, et al. Stapled α-helical peptide drug development: a potent dual inhibitor of MDM2 and MDMX for p53-dependent cancer therapy. Proc Natl Acad Sci U S A. 2013;110:E3445–3454. 23946421 10.1073/pnas.1303002110 PMC3767549

