# Supplementary material for: Synthetic lethality of combined ULK1 defection and p53 restoration induce pyroptosis by directly upregulating GSDME transcription and cleavage activation through ROS/NLRP3 signaling
Source: J Exp Clin Cancer Res. 2024 Aug 30;43:248. doi: 10.1186/s13046-024-03168-8 (PMC11363528; doi:10.1186/s13046-024-03168-8)
Supplement: Supplementary file 1 — Supplementary Material 1. [file 13046_2024_3168_MOESM1_ESM.docx]

**Supplementary Figure 1**


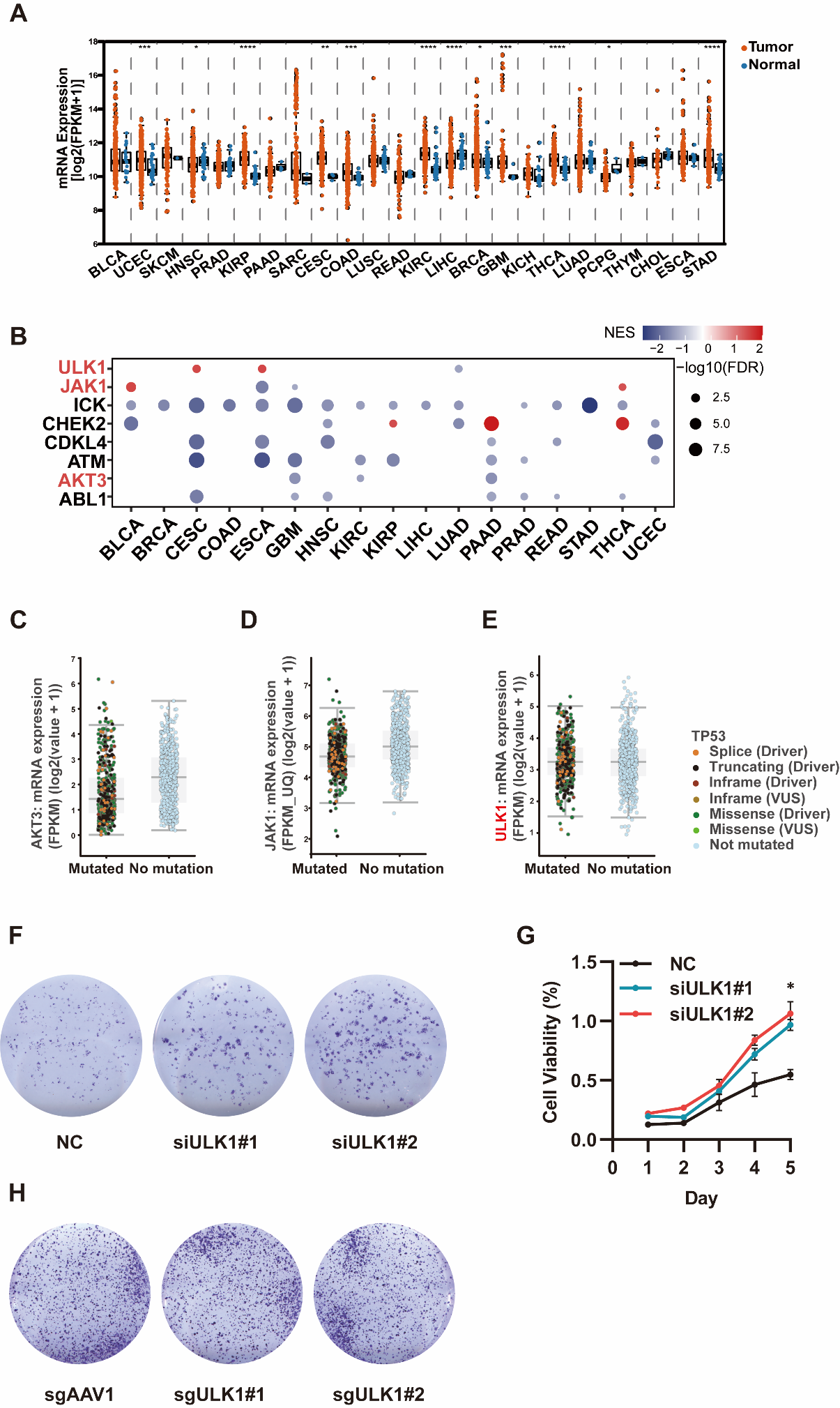
Figure S1: MDM2 inhibitor sensitivity in tumors varies widely. (**A**) MDM2 mRNA expression (log2(FPKM+1)) among all samples from the TCGA grouped by cancer. Each point represents one sample. The P values are based on two-tailed Student’s t test. (**B**) Enrichment analysis of the p53 signaling pathway between TCGA pancancer samples with high and low expression of the candidate genes. NES, normalized enrichment scores as determined by the GSEA algorithm. (**C-E**) Dot plot showing the differences in the mRNA expression of the indicated genes between patients with different TP53 mutation statuses. Each point represents one sample. (**F**) Colony formation assays were used to detect the proliferation of cancer cells after ULK1 knockdown by siRNA. (**G**) Cell viability was detected by MTT assays after ULK1 knockdown. (**H**) Colony formation assays were used to detect the proliferation of cancer cells after ULK1 knockout by sgRNA.

**Supplementary Figure 2**


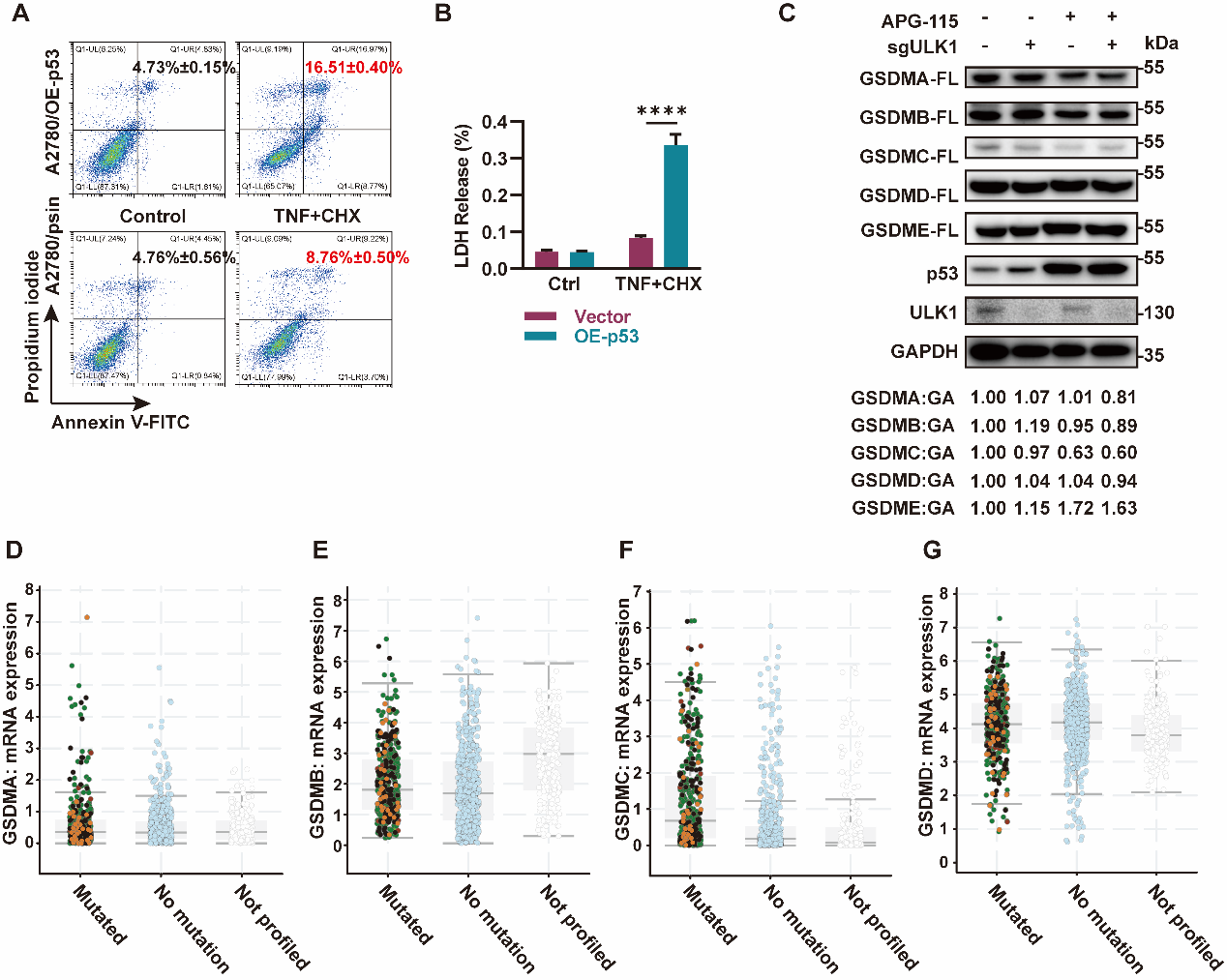


Figure S2: The transcription of only GSDME was directly regulated by p53. (**A-B**) Flow cytometric analysis of FITC staining, PI staining, and LDH release in A2780 p53-overexpressing cells following treatment with TNFα+CHX for 24 h. **** P < 0.0001. (**C**) Western blot showing GSDMs family protein levels in indicated cells. (**D-G**) Dot plot showing the differences in the mRNA expression of GSDME between patients with different TP53 mutation statuses. Each point represents one sample.

**Supplementary Figure 3**


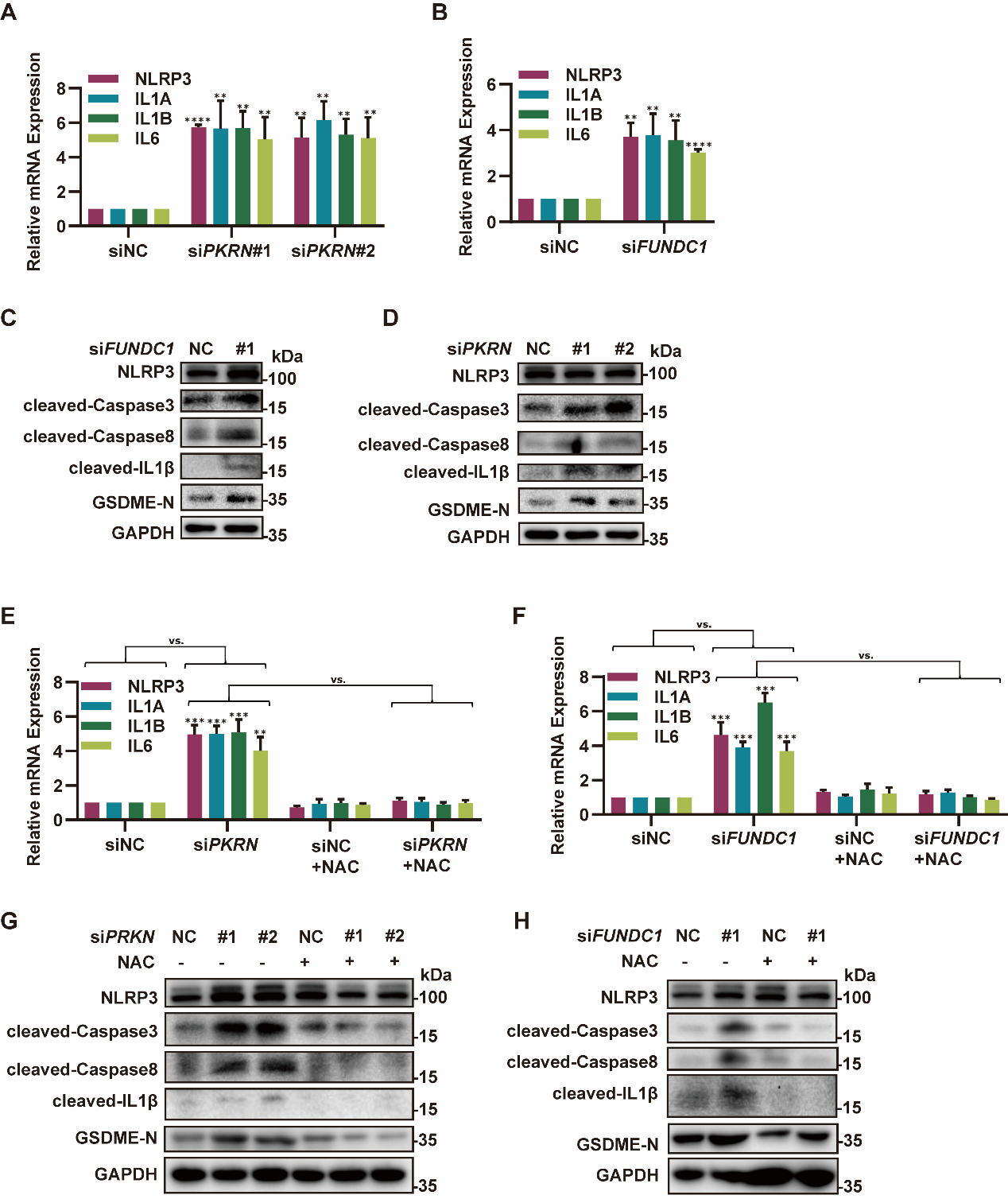
Figure S3: Mitophagy deficiency promote the activation of GSDME. (**A-B, E-F**) RNA was extracted from the indicated cells, and the expression of NLRP3, caspase 8 and IL1B was analyzed by qRT‒PCR. (**C-D, G-H**) Immunoblot analysis of NLRP3 pathway proteins and GAPDH from extracts of the indicated cells. (**E-H**) The indicated A2780 cells were treated with 4 mM NAC for 24 h.
